# Preparation and Characterization of Novel Perfluorooctyl Bromide Nanoparticle as Ultrasound Contrast Agent via Layer-by-Layer Self-Assembly for Folate-Receptor-Mediated Tumor Imaging

**DOI:** 10.1155/2016/6381464

**Published:** 2016-08-29

**Authors:** Yue Hu, Yong Wang, Jianshuai Jiang, Baosan Han, Shengmin Zhang, Keshi Li, ShuXiong Ge, Yahui Liu

**Affiliations:** ^1^Ningbo First Hospital, Ningbo Hospital of Zhejiang University, The First Affiliated Hospital of Ningbo Medical College of Ningbo University, Ningbo, Zhejiang 315010, China; ^2^Xin Hua Hospital Affiliated to Shanghai Jiao Tong University School of Medicine, Shanghai 200000, China; ^3^Ningbo Medical College of Ningbo University, Ningbo, Zhejiang 315010, China

## Abstract

A folate-polyethylene glycol-chitosan derivative was synthesized and its structure was characterized. An optimal perfluorooctyl bromide nanocore template was obtained via utilizing the ultrasonic emulsification method combining with orthogonal design. The targeted nanoparticles containing targeted shell of folate-polyethylene glycol-chitosan derivative and perfluorooctyl bromide nanocore template of ultrasound imaging were prepared successfully by exploiting layer-by-layer self-assembly as contrast agent for ultrasound. Properties of the novel perfluorooctyl bromide nanoparticle were extensively studied by Dynamic Light Scattering and Transmission Electron Microscopy. The targeted nanoparticle diameter, polydispersity, and zeta potential are around 229.5 nm, 0.205, and 44.7 ± 0.6 mV, respectively. The study revealed that spherical core-shell morphology was preserved. Excellent stability of targeted nanoparticle is evidenced by two weeks of room temperature stability tests. The results of the cell viability assay and the hemolysis test confirmed that the targeted nanoparticle has an excellent biocompatibility for using in cell studies and ultrasound imaging in vivo. Most importantly, in vitro cell experiments demonstrated that an increased amount of targeted nanoparticles was accumulated in hepatocellular carcinoma cell line Bel7402 relative to hepatoma cell line L02. And targeted nanoparticles had also shown better ultrasound imaging abilities in vitro. The data suggest that the novel targeted nanoparticle may be applicable to ultrasonic molecular imaging of folate-receptor overexpressed tumor.

## 1. Introduction

Recently, molecular imaging has received considerable attention and gradually becomes a hot research area in the field of disease diagnosis and treatment. It extends the diagnostic capability and utility of traditional imaging modalities [1–3]. In clinical practice, ultrasound imaging is applied more frequently to disease diagnosis. It has a lot of virtues such as being convenient in operation, noninvasive, nonradiative, flexible, and affordable [4, 5]. Therefore, in comparison with computed tomography (CT) and magnetic resonance imaging (MRI), ultrasonography has gained more acceptance by patients [6, 7]. With the emergence and development of ultrasound molecular imaging, the specificity and sensitivity of ultrasound imaging are expected to be improved greatly for various diseases [5, 8, 9]. As we all know, the fundamental enabling technology for ultrasonic molecular imaging is the contrast-enhanced ultrasound agents. Recently, commercially available ultrasound contrast agents (UCAs) such as SonoVue and Optison are widely used for ultrasound imaging, because they enhance the blood pool signal due to their large particle size (2–8 *μ*m). But they do not completely satisfy the requirements of ultrasound molecular imaging. Therefore, developing a more effective UCA for ultrasound imaging is part of the most urgent and important topics in ultrasound molecular imaging [10–12].

Tumor-specific ultrasound imaging has always been a problem to be resolved. Currently, researchers consider that an ideal UCA for molecular imaging of tumors should contain the following characteristics. First, UCA particles should be small enough to pass through the endothelial gaps of tumors (ranging from 380 to 780 nm) and achieve satisfactory extra vascular ultrasonic imaging. Second, the particles can be decorated with a specific functional group such as antibody, transferrin, cyclic pentapeptide (cRGD), and folic acid so that they can be actively targeted to specific tumor tissues for uptake into tumor cells. These characteristics should facilitate effective contrast for ultrasonic imaging of tumor cells [8, 11, 13, 14].

Over the past several years, there has been a rapid growth in a multitude of available nanosized contrast agents for tumor imaging [10, 15]. Among them, UCAs made by liquid perfluorocarbons (PFCs) show many unique advantages such as chemical inertness and stability, more safety and biocompatibility, and efficient ultrasound contrast enhancement [16, 17]. Although there are many reports on liquid perfluorocarbon nanoparticles with mean diameter of several hundred nanometers, active targeting delivery to a specific tissue is still one of the obstacles. An elegant approach to increase delivery efficacy is to link ligands to the nanoparticle surface via chemical modification for tissue targeting through cellular receptor [17, 18]. However, there were certain deficiencies in these studies, such as complicated preparation processes, significant increase of particle size, and undesirable effects of ultrasound imaging [19]. Nevertheless, these studies reported that folate had shown great potential as a targeting ligand, displaying nonimmunogenicity, infinite availability, defined conjugation chemistry, and a favorable nondestructive cellular internalization pathway via binding to the folate receptor (FR). FR is highly overexpressed in various types of human tumors, including liver cancer, but generally absent on the surface of normal cells [14, 20, 21]. In this paper, we obtain a FA-polyethylene glycol- (PEG-) chitosan (CS) derivative by chemical reactions and perfluorooctyl bromide nanocore template using the ultrasonic emulsification method. Our strategy is to bind FA-PEG-CS to nanocore template through electrostatic interactions, promoting the formation of a new FR-mediated perfluorooctyl bromide nanoparticles via a layer-by-layer (LbL) assembling technique. Subsequently, we design different experiments to evaluate the physicochemical characteristics of the formulations. In addition, the in vitro targeting of the nanoparticles was measured and compared using normal liver cell L02 (FR low-expression) and liver cancer cell Bel7402 (FR overexpression).

## 2. Materials and Methods

### 2.1. Materials

Perfluorooctyl bromide (PFOB), folic acid (FA), and 1-(3-dimethylaminopropyl)-3-ethylcarbodiimide hydrochloride (EDC) were provided by Sigma-Aldrich (USA), LutrolF68 (F68) was obtained from BSAF (Germany), egg lecithin (PL-100M) was provided by AVT (Japan), chitosan (CS) (m.w. 25–50 kg/mol, degree of deacetylation 95%) was obtained from Haidebei Marine Bioengineering Co., Ltd. (China), folic acid PEG acid (FA-PEG-COOH) (m.w. 3400 g/mol) was supplied by Nanocs (USA), glycerol was purchased from Hunan Erkang Pharmaceutical Co. Ltd. (China), folic acid was provided by Aladdin (China), fluorescein isothiocyanate (FITC) was obtain from Solarbio (China), N-hydroxysuccinimide (NHS) and N,N′-dicyclohexylcarbodiimide (DCC) were supplied by Sinopharm Chemical Reagent Co., Ltd. (China), the dialysis membrane (MwCO = 8000–14000) was provided by Sigma-Aldrich (USA), Spectra/Gel Absorbent was obtained from spectrum (USA), water was purified using system from Millipore (France), and other chemicals and solvent were of analytical grade. Trypsin, fetal bovine serum (FBS), RPMI1640, and folate-free RPMI1640 media were purchased from Gibco (USA), cells were supplied by ATCC (USA), and CellTiter96® Aqueous One Solution Cell Proliferation Assay (MTS) was obtained from Promega Corporation (USA).

### 2.2. Synthesis of FA-PEG-CS Conjugate and Labeling of FA-PEG-CS with FITC

**Figure 1:**
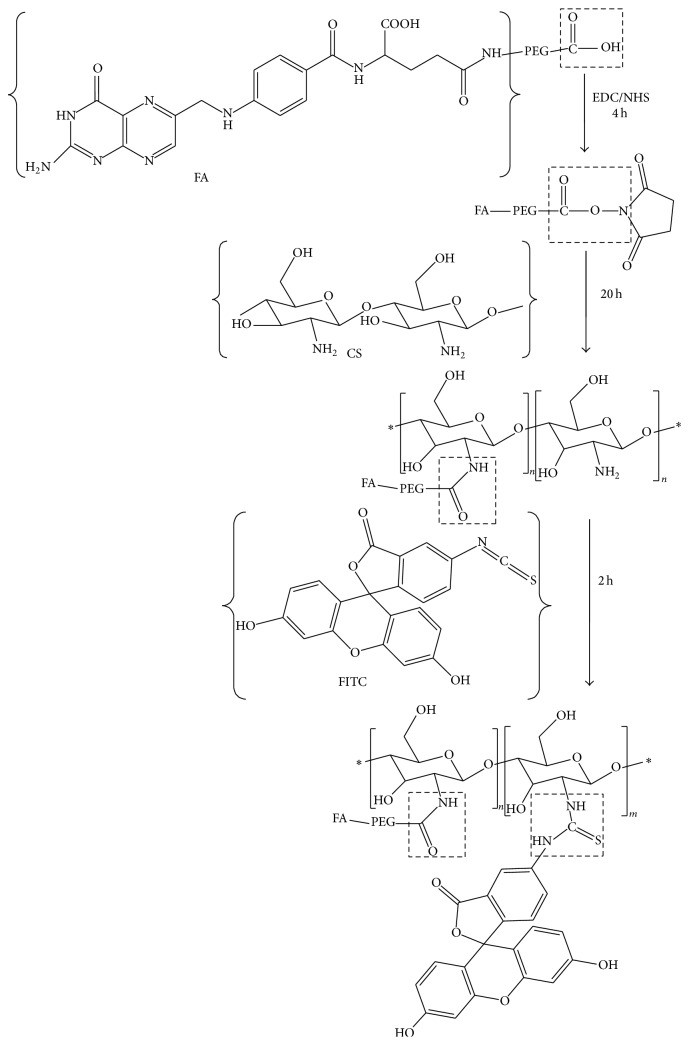
The modification process of chitosan derivatives. ∗ represents a number of repeating groups.

Synthetic scheme of FA-PEG-CS is shown in Figure 1. FA-PEG-COOH conjugation to CS was carried out by EDC/NHS chemistry [22]. Briefly, FA-PEG-COOH (40 mg) dissolved in 5 mL of DMSO was activated with EDC (2.5 mg, 15 *μ*mol) and NHS (5.8 mg, 25 *μ*mol) at room temperature for 4 h. 20 mL of 2% acetic acid aqueous solution was added dropwise to FA-PEG/DMSO solution containing EDC and NHS and stirred for 20 h at room temperature in the dark. The reaction product was dialyzed by using a dialysis membrane (molecular weight cut-off 8–10 kDa) against DMSO for 3 days to remove unreacted FA-PEG and then purified against distilled water for 3 days to remove DMSO; after that the reacted product was lyophilized.

FA-PEG-CS (100 mg) dissolved in 20 mL of 2% acetic acid aqueous solution was then labeled with FITC (10 mg) dispersed in 10 mL of ethanol in the dark for 2 h, with constant stirring. After labeling, the product was dialyzed using a dialysis membrane (molecular weight cut-off 1 kDa) against distilled water for about 2 days to remove unreacted FITC, followed by freeze-drying in the dark.

### 2.3. Structural Characterization of FA-PEG-CS Conjugate and FA-PEG-CS-FITC

Fourier transform infrared (FTIR) spectra of FA-PEG-COOH and FA-PEG-CS were recorded with KBr pellets using a Nicolet 6700 series spectrometer (Thermo, USA). 1H nuclear magnetic resonance (1H NMR) spectra of FA-PEG-COOH dissolved in DMSO and FA-PEG-CS conjugate dissolved in DCl/D2O (1 : 100, v/v) mixture were measured on AVANCE III 400 MHZ spectrometer (Bruker, Germany) at room temperature.

### 2.4. Formulation Optimization of PFOB Nanocore Template

PFOB nanocore templates were prepared by ultrasonic emulsification method [23, 24]. Briefly, LutrolF68, egg lecithin, and oleic acid were chosen as emulsifiers and stabilizer agent, respectively. Glycerol and PFOB were used as the osmotic pressure regulator and oil phase, respectively. Glycerol (2.25% (w/v)) was dissolved in purified water as the water phase. LutrolF68, oleic acid, and egg lecithin were mixed in 4 mL of water phase by vortex. Then the mixture was incubated at 37°C in a shaking incubator for 1 h. PFOB was added dropwise to water phase containing the above-mentioned substances and stirred for 5 min by vortex at room temperature. Finally, the solution was sonicated to form the PFOB nanocore template. An L9(34) orthogonal design was established to optimize nanocore template, as showed in the Tables 1 and 2, using size, charge, and PDI as the indexes. The physicochemical properties of the formulations including size, charge, shape, and morphology were investigated using Dynamic Light Scattering (DLS) and Transmission Electron Microscopy (TEM).

### 2.5. Preparation and Characterization of Targeted Nanoparticle through Layer-by-Layer Self-Assembly

A dispersion solution of 5% w/v FA-PEG-CS-FITC in 1% v/v acetic acid aqueous solution was adjusted to pH 6.0 by using 1 M NaOH. PFOB nanocore templates were added dropwise in the above solution with magnetic stirring. The two substances were continuously stirred for another 1 h and incubated for 30 min at 37°C to ensure sufficient conjugation. The mixture was centrifuged at 15000 rpm for 10 min at 4°C. The supernatant was discarded and the pellet was washed three times using purified water. The pellet was dispersed in 5% w/v mannitol aqueous solution by low-frequency ultrasound and filtrated by the 0.45 *μ*m membrane filtration to remove large impurities. Size and zeta potential of nanoparticle were measured by NanoSizer measurement based on Dynamic Light Scattering (DLS) principle at 25°C, along with pH values recorded. The nanoparticle was deposited onto a Formvar-coated copper grid and stained with a 1% uranyl acetate solution. Morphologies were investigated with TEM.

### 2.6. Stability of Targeted Nanoparticle

Physicochemical stability of targeted nanoparticle was characterized in long term at room temperature. The stability parameters including size, zeta potential, PDI, and pH were determined as function of storage time by DLS and pH meter at days 0, 7, and 15.

### 2.7. Cell Viability Assay

Human hepatoblastoma cells Bel7402 and normal human hepatocellular L02 were cultured in RPMI1640 medium without folate, supplemented with 10% fetal bovine serum, 1% streptomycin, and penicillin. In vitro cell viability tests were performed using the Cell Titer 96 Aqueous One Solution Cell Proliferation Assay [25]. Briefly, Bel7402 and L02 were seeded in 96-well plates at density of 1 × 10^4^ cells/well. All cells were incubated in 0.2 mL of growth medium without folate at 37°C in humidified 5% CO_2_ atmosphere for 24 h. Targeted nanoparticle concentration was adjusted to 5.58, 2.79, 1.40, 0.7, and 0 nM by using RPMI1640 medium without folate, supplemented with 10% fetal bovine serum, 1% streptomycin, and penicillin. After incubation for 24 h, growth medium was replaced by 0.2 mL fresh folate-free medium containing various concentrations of nanoparticle and cells were cultured for a further 24 h. The medium was removed and the adhered cells were washed three times with PBS. The medium containing 20 *μ*L of Cell Titer 96 Aqueous One Solution Reagent was added into each well. After further incubation for 1 h, absorbance was measured at 490 nm and 630 nm using a microplate reader.

### 2.8. Erythrocyte Hemolysis Test

A blood sample was obtained from a healthy human by venipuncture and was collected in a test tube containing heparin sodium. The erythrocytes were immediately isolated from whole blood by centrifugation at 2000 ×g for 5 min and washed three times with five volumes of normal saline solution. The collected erythrocyte (1 mL) was resuspended in 50 mL of normal saline. Targeted nanoparticle concentration was adjusted to 5.58, 2.79, 1.40, and 0.7 nM by using normal saline solution. Aliquots (0.3 mL) of targeted nanoparticle at different concentrations were added to 2.5 mL of 2% erythrocyte solution, and normal saline was added to a final volume of 5 mL. Incubation was carried out at 37°C for 3 h. Then, the samples were centrifuged at 2000 ×g for 5 min, with 0.4 mL of the resulting supernatant diluted to 5 mL with (99 : 1) ethanol/HCl mixture. The absorbance of the mixture was measured at 398 nm to determine the percentage of hemolysis. Hemolysis induced with water and normal saline was taken 100% and 0% [24].

### 2.9. In Vitro Tumor-Targeting Ability of Targeted Nanoparticle

To confirm folate-receptor-mediated intracellular uptake of targeted nanoparticle, competition assay using fluorescence microscope and flow cytometry were done with cultured Bel7402 and L02 cells in RPMI1640 with or without folate. Cells were seeded in 6-well plates at an initial density of 5 × 10^5^ cells/well of folate-free growth medium for 24 h. Bel7402 and L02 cells were divided into two groups that the medium was replaced with 2 mL fresh free-folate growth medium plus 100 *μ*L PBS and 100 *μ*L targeted nanoparticle, respectively. The two groups were incubated for a further 2 h. After incubation, cells were washed three times with PBS to remove nanoparticle not taken up by cells present in the medium. Anhydrous ethanol/acetone (1 : 1) was added to cell to fix for 15 min at −20°C and was washed with PBS. Then, cells associated with fluorescence were imaged by a fluorescence microscope.

Flow cytometry was used to further quantify the folate-receptor-mediated specificity of targeted nanoparticle. Bel7402 and L02 cells were cultured in 12-well plate at a density of 2 × 10^5^ cells/well for 24 h and then allowed to grow for 2 h with 100 *μ*L PBS and 100 *μ*L targeted nanoparticle in 1 mL free-folate medium, respectively. After that, the media were aspirated and the cells were washed three times with PBS. Trypsin (1 mL) was added to each well for 5 min and then the cells were collected, centrifuged at 1000 ×g for 5 min, fixed by anhydrous ethanol/acetone (1 : 1) for 15 min at −20°C, and resuspended in 0.5 mL PBS. The intracellular FITC was quantified using flow cytometry.

### 2.10. In Vitro Ultrasound Imaging

To characterize the ultrasonic imaging ability of the targeted nanoparticle, in vitro ultrasound experiment was performed. The targeted nanoparticle was adjusted to various concentrations including 14.2, 7.10, 3.55, and 1.78 nM by using purified water. 2 mL of targeted nanoparticle at above concentration was added to the Latex gloves. Positive and negative controls were prepared by the addition of 2 mL SonoVue and normal saline to the Latex gloves. Clinical ultrasound scanner system was used to obtain ultrasonic contrast image of the samples by setting superficial organs routine. The probe frequency was 3–11 MHz, the mechanical index was 0.7, and the gain was 60%.

## 3. Results and Discussion

### 3.1. Synthesis of FA-PEG-CS Conjugate and Labeling of FA-PEG-CS with FITC

**Figure 2:**
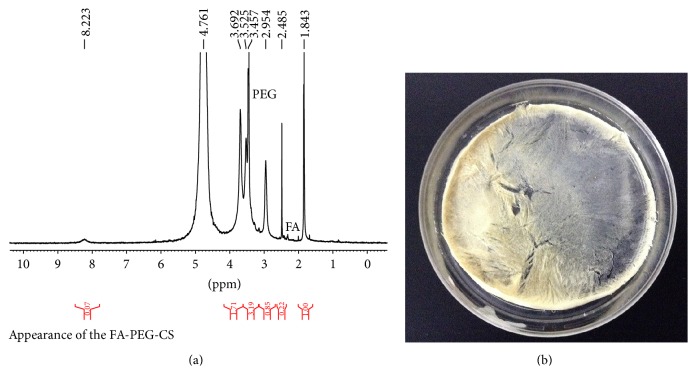
(a) 1H NMR spectrum of FA-PEG-CS. Solvent DCl/D2O (1 : 100). (b) Physical appearance of the FA-PEG-CS.

**Figure 3:**
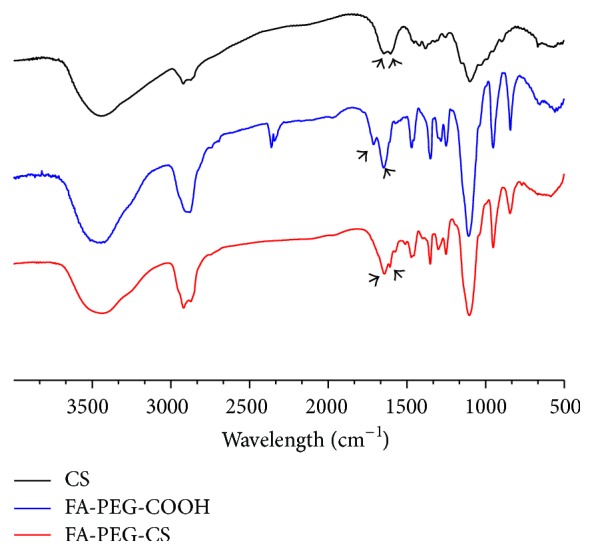
FT-IR spectra of CS (top), FA-PEG-COOH (middle), and FA-PEG-CS (bottom).

FA-PEG-CS was successfully synthesized as showed in Figures 1 and 2(b). It should be noted that only 25 and 50 kDa chitosan are able to conjugate with FA and the folate content was confirmed to be 0.9%–1.1 mol% for 25–50 kDa FA-PEG-CS with respect to chitosan glucosamine units [22]. FA-PEG-CS was synthesized by the reaction of carboxylic acid group of FA-PEG and the amino groups of CS through amide linage [22, 25, 26]. The composition of the synthesized conjugate was analyzed by 1H NMR and FTIR spectra, respectively, as showed in Figures 2(a) and 3. The conjugation of FA-PEG-CS was verified by comparing the 1H NMR spectra with a previous study. The molar grafting ratio of FA-PEG and CS was about 1.3–4 folate molecules on each of the 25–50 kDa CS-PEG-FA chains as determined by 1H NMR [22]. In FTIR spectra (Figure 3) and the peaks at 1091 cm^−1^ were assigned to the bending mode of -(CH_2_-CH_2_)- in PEG, which proved successful attachment of PEG onto CS. Peaks at 1643 cm^−1^ and 1568 cm^−1^ were assigned to stretching modes of amide C=O and N-H groups, respectively, suggestive of successful attachment of FA-PEG onto CS. The above-mentioned specific peaks are consistent with a previous work [27].

### 3.2. Formulation Optimization of PFOB Nanocore Template

**Figure 4:**
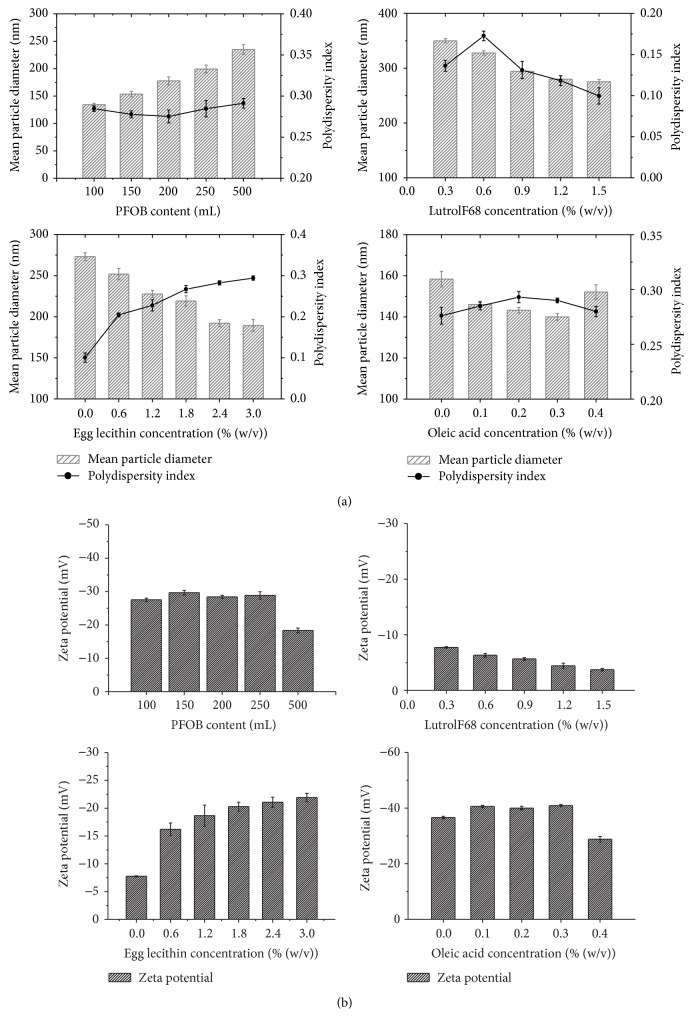
(a) Effect on particle diameter, polydispersity index of varying of the content of PFOB, the concentration of LutrolF68, the concentration of LutrolF68, and the concentration of oleic acid; (b) effect on particle diameter and zeta potential of varying of the content of PFOB, the concentration of LutrolF68, the concentration of LutrolF68, and the concentration of oleic acid.

Firstly, four factors including PFOB, LutrolF68, folic acid, and egg lecithin were prescreened by varying only one factor at a time. Prior to orthogonal design, the levels of the relevant factors were identified by univariate analysis. The influences of PFOB, LutrolF68, egg lecithin, and oleic acid on average diameter, zeta potential, and PDI are shown in Figure 4. The average particle sizes of each sample decreased with increasing concentrations of LutrolF68, egg lecithin, and oleic acid and with decreasing concentrations of PFOB. The zeta potential was increased with the concentrations of egg lecithin and oleic acid. PDI was found to slightly increase with the concentration of less than 0.3% LutrolF68. When the concentration of LutrolF68 exceeded 0.3%, PDI will be decreased. However, PDI was increased apparently with egg lecithin levels.

The results of the orthogonal design study are shown in Table 3. The concentrations of PFOB, LutrolF68, folic acid, and egg lecithin were chosen as the most influential factors. Based on the enhanced permeability and retention (EPR) effect, the only particles whose size was smaller than the endothelial gaps of tumors (ranging from 380 to 780 nm) achieve passive-targeting strategy, so the particle size that was the most important factor in the process of preparing nanoparticles needs to be considered [11]. Taking particle size as one of the evaluated indexes, the four factors were analyzed at three different levels.* K*_1_,* K*_2_, and* K*_3_ were the average diameter of level 1, level 2, and level 3 for each factor. The level with the smallest size was considered as the optimal level of each factor. For all the experiments in the orthogonal design, the average diameter was within a range of 130–183 nm. The ranking of the four factors in this experiment was PFOB (A) > LutrolF68 (C) > egg lecithin (B) > folic acid (D), and the individual levels within each factor were ranked as PFOB, 1 < 2 < 3; LutrolF68, 2 < 3 < 1; egg lecithin, 3 < 2 < 1; and folic acid, 2 < 3 < 1. The formulation of optimal size was obtained to be A1 B3 C2 D2. The polydispersity index represented the uniformity of the particles. The value of PDI was larger, the distribution of particle size was wider, and the homogeneity was poor [28]. In our study, the PDI was within a range of 0.259–0.293 nm. The ranking of the four factors in this experiment was PFOB (A) > LutrolF68 (C) > egg lecithin (B) > folic acid (D). The formulation of optimal PDI was A3B2C1D3. The zeta potential was within a range of −34.8–57.8 nm. The ranking of the four factors in this experiment was LutrolF68 (C) > egg lecithin (B) > PFOB (A) > folic acid (D). The formulation of optimal PDI was obtained to be A1B1C1D3. The PFOB nanocore template needs to meet the requirement of layer-by-layer self-assembly; therefore it had smaller average particle size, better dispersion, and more sufficient zeta potential. The most appropriate PFOB nanocore template prescription in our research was considered to be A1B2C2D3 by comprehensive analysis of the above-mentioned three indicators.

Secondly, four factors were investigated by changing only one factor at a time. The influences of ultrasonic intensity (A), ultrasonic time (B), ultrasonic cycles (C), and temperature (D) on the average diameter, zeta potential, and PDI are shown in Table 4. We identified the influential factors of optimal PFOB nanocore template prescription: 40% ultrasonic intensity, 10 min, pulses of 10 s, with a pause of 10 s 20 between pulses, and 25 ± 4°C.

**Figure 5:**
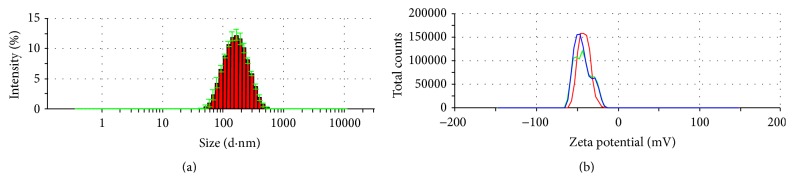
The particle size distribution (a) and zeta potential (b) spectrum of optimal PFOB nanocore template.

**Figure 6:**
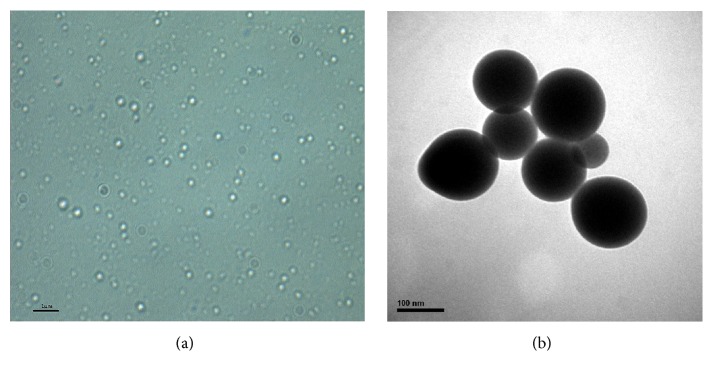
Optical microscopy (a) and TEM (b) image of optimal PFOB nanocore template.

The average particle size of optimal PFOB nanocore template was 131.5 ± 4.3 nm. The particle diameter at 93.5% of the cumulative particle size distribution was less than 300 nm. Polydispersity index and zeta potential are 0.265 ± 0.006 and −42.8 ± 1.0 mV. pH of optimal PFOB nanocore template was confirmed to be 6.73 ± 0.11. This pH was determined to meet the requirements of intravenous emulsion. The particle size and zeta potential distribution spectrum for optimal PFOB nanocore template are shown in Figure 5. Images are shown in the Figure 6. Morphological analysis was carried out by optical microscopy and Transmission Electronic Microscopy imaging, which observed spherical appearance of objects with diameters which were in good agreement with the particle size determined as described above.

Numerous studies have demonstrated that emulsification and ultrasonic method is widely used to prepare liquid nanoemulsions [23, 29]. Since it is time-saving and convenient and allows control of the particle diameter, it was used in this research to prepare PFOB nanocore template. The previous studies showed that the emulsion size, zeta potential, and stability were closely related to surfactant and drug. Egg lecithin is usually employed as emulsifier in parenteral emulsions and liposomes, due to its safety. The oxidation index of egg lecithin PL-100M was lower than 0.2 meeting the requirement of the oxidation index of phospholipids in Chinese Pharmacopoeia, 2010 [30]. In the present study, phospholipid can be deposited on the surface of drug-filled nanoemulsions as a single monolayer. Such structure may provide a protective interface and highly negative charges to prevent the fusion of nanodroplets [31, 32]. Based on an overall consideration of various factors, PL-100M was the optimum phospholipid for the preparation of PFOB nanocore template. However, single use of an emulsifier has several limitations, such as requiring a large amount of surfactant and high-energy emulsification method. Currently, some researches have shown that the combination of various emulsifiers can bring better results. The composite application of emulsifiers cannot only reduce the amount of emulsifiers and toxicity and decrease the particle size, as well as increase the strength of the interfacial film [33, 34]. It has been reported that due to the nonionic surfactants pluronic F68 has good behavior in blood systems, which have been used as nanoemulsion for drug and gene delivery [35]. F68 interacting with phospholipid on the surface of emulsions form an expanded film-like surface behavior. This structure and stability of the mixed system may be much better than the system with phospholipid alone [32]. Adding F68 to the interface led to a decrease in the zeta potential, which increased the flexibility of the film. Oleic acid is a preferred stabilizer for preparing the emulsion. Additional small amount of oleic acid can enhance the interaction between F68 and phospholipid, which increase the zeta potential and enhance the stability [36, 37]. Therefore, this study selected PL-100M and F68 as emulsifiers, oleic acid as stabilizer, and PFOB as core drug that successfully prepared PFOB nanocore template.

Ultrasonic emulsification method is a common and convenient method for preparing nanoemulsions. Tremendous shear and cavitation generated by ultrasound dispersed larger particle into nanoparticles. Studies have shown that the shear force and cavitation can be upregulated with the increase of the intensity of ultrasound to produce sufficiently small particles. This result is consistent with our above-mentioned research [29, 38]. The past studies have demonstrated that the diameter of the emulsion particles gradually decreased with the increase of ultrasonic time, but we found that the particle size did not significantly change after ultrasonic time more than 20 min and prolonged ultrasonic time will increase temperature. The nature of the drugs and excipient was destroyed by high temperature which would affect emulsifying effect at some extent. Therefore, ultrasonic preparation determined in our study is more suitable for optimal PFOB nanocore template.

### 3.3. Layer-by-Layer Self-Assembled Targeted Nanoparticle Preparation and Characterization

**Figure 7:**
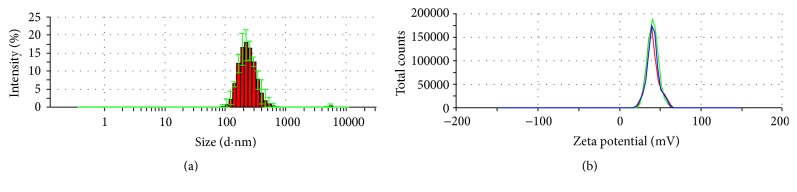
The particle size distribution (a) and zeta potential (b) spectrum of targeted nanoparticle.

**Figure 8:**
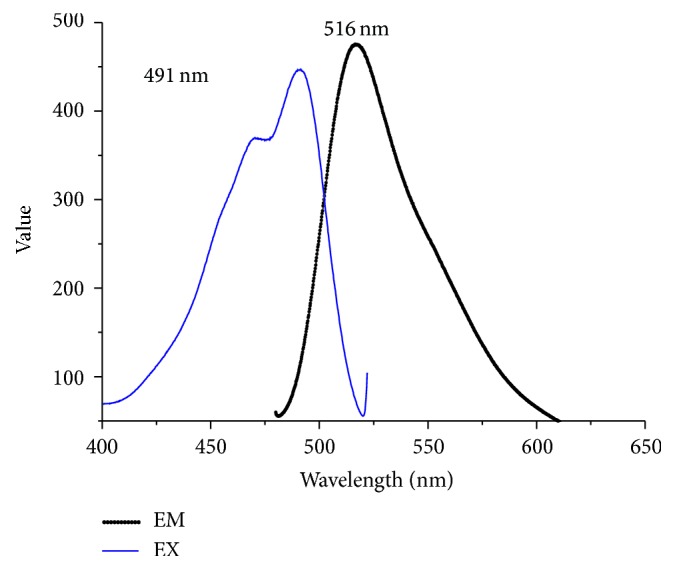
The fluorescence spectrum of targeted nanoparticle.

**Figure 9:**
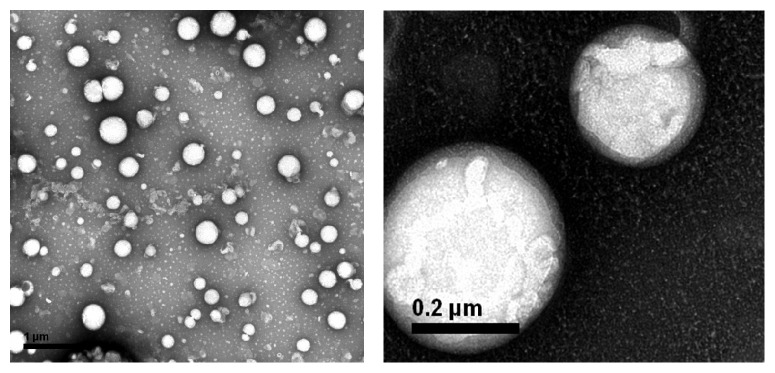
TEM image of targeted nanoparticle.

The layer-by-layer assembly technology is based on the electrostatic interactions between various polyelectrolytes with opposite charges [39, 40]. To avoid the aggregation, PFOB nanocore template dispersion solution was dropped into FA-PEG-CS-FITC acetic acid aqueous solution with gentle magnetic stirring. In this procedure, the PFOB nanocore templates were independent, and each of them would be coated by FA-PEG-CS-FITC immediately. As a result, the outside layer of the PFOB nanocore templates was covered with FA-PEG-CS-FITC as positive charge, which could inhibit the aggregation among preparing targeted nanoparticles through the electrostatic repulsive forces. After sufficient incubation of PFOB nanocore templates in FA-PEG-CS-FITC solution to achieve complete surface coverage, the targeted nanoparticles were centrifuged and washed to remove the free FA-PEG-CS-FITC. Finally, we obtained purified targeted nanoparticle. As showed in Figure 7, the average diameter showed 229.5 ± 7.5 nm, values between 91.28 nm and 615.1 nm. The particle diameter at 92.6% of the cumulative particle size distribution was less than 500 nm. Polydispersity index and zeta potential are 0.205 ± 0.014 and 44.7 ± 0.6 mV. pH was confirmed to be 6.8 ± 0.09. Based on the previous studies [28], values of PDI between 0.1 and 0.25 showed a narrow distribution of particles, and the absolute value of zeta potential greater than 30 mV shows good stability of particles. The targeted nanoparticle of this study was in full compliance with the parameters reported in previous paper.  Figure 8 showed that the fluorescence of FITC was measured in targeted nanoparticle by using fluorescence spectrophotometer. In the picture, the targeted nanoparticles were obtained by excitation peak at 491 nm and emission peak at 516 nm, which was similar to fluorescence spectrum of FITC, which confirmed that FA-PEG-CS-FITC was successful combined with the surface of PFOB nanocore template. As showed in Figure 9, TEM imaging of targeted nanoparticle further indicated the morphology and the particle size. The targeted nanoparticle had about 200 nm spherical appearance. The PFOB core appears in gray, whereas the FA-PEG-CS-FITC shell seems transparent film. The average particle sizes obtained by DLS were in good agreement with the sizes observed by TEM.

The targeted nanoparticle for biomedical applications should be small, uniform, and stable [41]. To determine the stability of targeted nanoparticle over an extended period of time, long-term room temperature stability tests were performed, as described in Table 5. The results show that although the mean particle size of seven days increased by about 10 nm (*p* < 0.05), there were minimal changes compared to seven days and fifteen days (the difference was not statistically significant, *p* < 0.05). PDI and pH were also no significant variation for about two weeks, implying that targeted nanoparticle was of good stability for clinical applications.

### 3.4. Biocompatibility Tests

**Figure 10:**
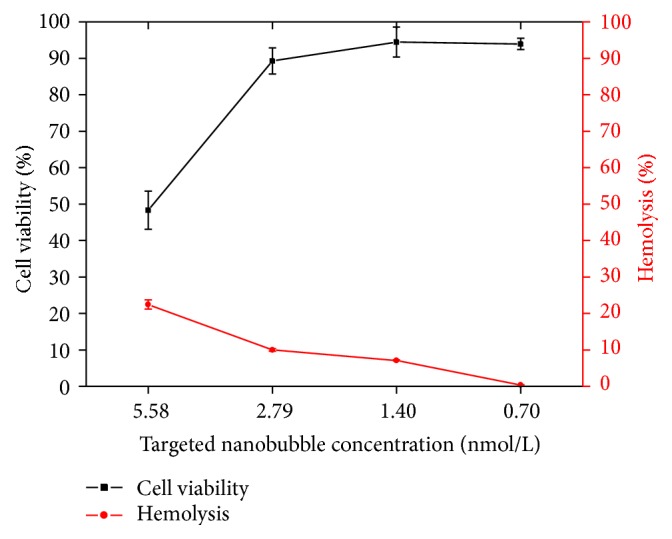
The results of targeted nanoparticle biological safety. In vitro tumor-targeting ability of targeted nanoparticle.

To explore the effect of targeted nanoparticle on cellular toxicity, studies were performed by incubating nanoparticle with L02 cells at different concentrations after 24 h. The cell viability results are shown in Figure 10. Cell toxicity was decreased with decreasing concentrations of nanoparticle. The targeted nanoparticle showed high cell viability about 89.5% even at higher concentration of 2.79 nmol/L. It was found that the nanoparticle had lower effect on cytotoxicity, and particularly it had no toxicity to normal liver cells under the concentration less than 2.79 nmol/L in vitro.

To use nanoparticle for intravenous administration and evaluate the safety of nanoparticles themselves, in vitro erythrocyte hemolysis tests were carried out [42]. As showed in Figure 10, the percentage of hemolysis induced by targeted nanoparticle reduced with concentration. When the concentration of targeted nanoparticle was less than 2.79 nmol/L, the percentage of human erythrocytes undergoing hemolysis was lower than 10%. Therefore, the presence of lower concentrations of nanoparticle had no effect on the rate of hemolysis.

The results of the cell viability assay and the hemolysis test, which were used to determine the cytotoxicity of nanoparticle, confirmed its biological safety. The above results confirmed that the targeted nanoparticle may be of excellent biocompatibility for using cell studies and ultrasound imaging in vivo.

**Figure 11:**
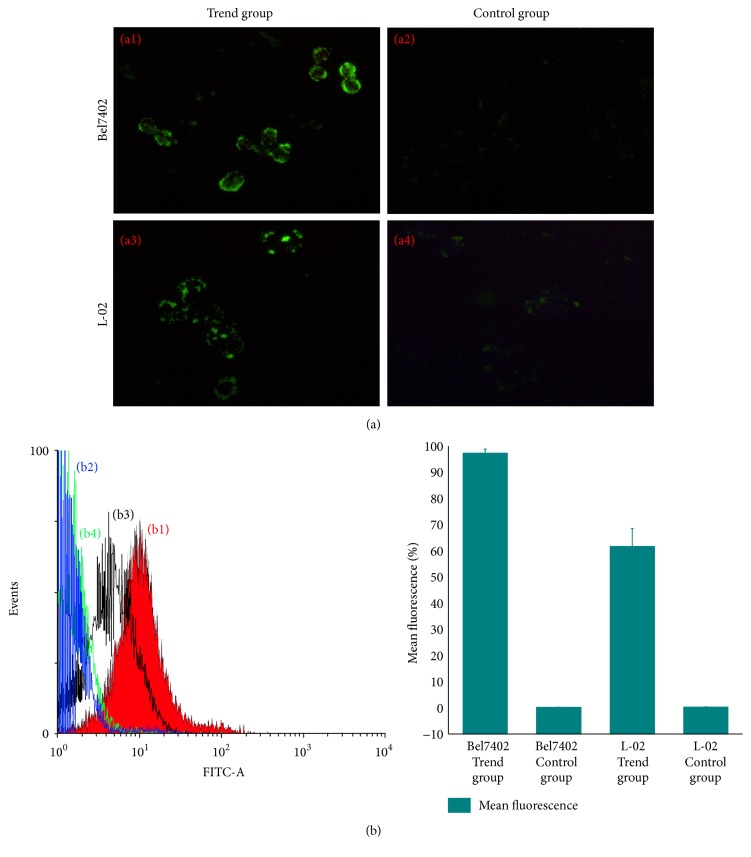
Fluorescent microscopic images and flow cytometry histogram of cell uptake. (a) Flurorescent microscopic images of cell uptake. (b) Fluorescence-activated cell sorting analysis of uptake: (b1) Bel7402 of trend group; (b2) Bel7402 of control group. (b3) L-02 of trend group and (b4) L-02 of control group.

To determine whether targeted nanoparticle can be internalized by FR overexpressed tumor cells, FITC fluorescence of the targeted nanoparticle allowed the direct visualization of nanoparticle uptake by Bel7402 and L02 cells. The fluorescence image of Bel7402 cells after 2 h incubation in the presence of targeted nanoparticle is shown Figure 11(a), in which intensive fluorescence was clearly observed. It indicated that events of significant cell uptake of targeted nanoparticle happened. On the contrary, fluorescence of L02 cells was remarkably weaker when the cells were incubated for 2 h in the presence of targeted nanoparticle. The intracellular uptake of the control group by Bel7402 and L02 is almost negligible. The above results demonstrated that the intracellular uptake of targeted nanoparticle by hepatoma cell line Bel7402 of FR overexpression is very strong; however that of targeted nanoparticle by hepatoma cell line L02 of FR less expression is less efficient.

As showed in Figure 11(b) with a similar tendency as for flow cytometry, 97.3 ± 1.55% Bel7402 incubated with targeted nanoparticles showed fluorescence uptake. In comparison, only 61.57 ± 6.96% (*p* < 0.05) L02 with targeted nanoparticles showed fluorescence uptake. 0.15 ± 0.05% Bel7402 and 0.23 ± 0.13% L02 showed little fluorescence uptake in the control group. The results indicate that greater intracellular uptake of targeted nanoparticles via FR mediation was observed in hepatoma cell line in vitro condition.

### 3.5. In Vitro Ultrasound Imaging

**Figure 12:**
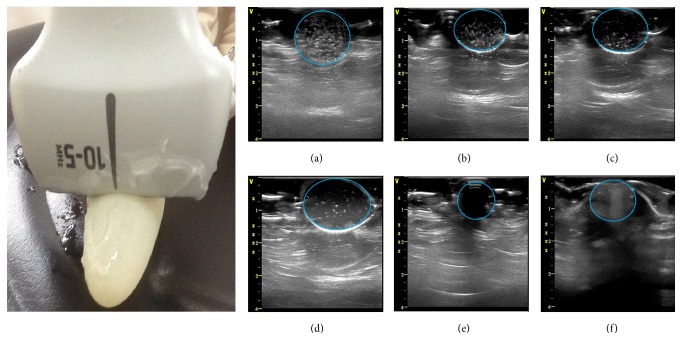
In vitro ultrasound imaging of different concentration the targeted nanoparticles, respectively, concentration of 14.20 (a), 7.10 (b), 3.55 (c), and 1.78 (d) nmol/L in distilled water, distilled water as negative control (e), and commercially available contrast agent SonoVue as positive control (f).

In order to evaluate ultrasonic behavior of the targeted nanoparticles, ultrasound images were obtained at various nanoparticle concentrations using diagnostic frequency ultrasound. As showed in Figure 12, it is observed that with the nanoparticle concentration increased the ultrasonic signals of the nanoparticle raised. Nanoparticle showed better ultrasonic contrast enhancement ability in gray-scale intensity. Based on the experimental results, we predict that the targeted nanoparticles are more promising for targeted tumor ultrasound imaging.

## 4. Conclusions

Tumor molecular imaging is an important topic; based on requirement of tumor molecular imaging by ultrasound, we have successfully designed and prepared a good biocompatible targeted nanoparticle ultrasound contrast agent and evaluated its tumor-targeting and ultrasound imaging ability in vitro. In this work, PFOB nanocore template was prepared and optimized via using ultrasonic emulsification method. The synthesized FA-PEG-CS conjugate as targeting shell material was coated on the surface of PFOB nanocore template by layer-by-layer self-assembly technique. We found a pathway toward the development of novel targeted nanoscale contrast-enhanced ultrasound imaging agent. The fabricated targeted nanoparticles have illustrated better tumor-targeting and ultrasound imaging ability in vitro. Their characteristics suggest that the novel targeted nanoparticle may be applicable to ultrasonic molecular imaging of FR overexpressed tumor. Further studies will focus on the investigation of biocompatible and the potential ultrasound imaging ability in vivo using tumor-bearing animal models.

## Figures and Tables

**Table 1:** Factors and levels of orthogonal design.

Factors	PFOB (*μ*L)	Egg lecithin (%, w/v)	LutrolF68 (%, w/v)	Oleic acid (%, w/v)
Level 1	100	1.8	0	0.1
Level 2	150	2.4	0.6	0.2
Level 3	200	3.0	1.3	0.3

**Table 2:** Composition of the PFOB nanocore templates.

Groups	Factors			
	PFOB (*μ*L)	Egg lecithin (%, w/v)	LutrolF68 (%, w/v)	Oleic acid (%, w/v)
	A	B	C	D
1	3	2	3	1
2	3	3	1	2
3	2	1	3	2
4	2	3	2	1
5	2	2	1	3
6	1	3	3	3
7	1	1	1	1
8	3	1	2	3
9	1	2	2	2

**Table 3:** Results of orthogonal design: the four factors were analyzed at three different levels. *K*_1_, *K*_2_, and *K*_3_ were the average diameter of level 1, level 2, and level 3 for each factor.

Groups	A	B	C	D	The average diameter *X* (nm)	PDI *Y*	Zeta potential *Z* (mV)
1	200	2.4	1.2	0.1	182.7	0.262	−34.8
2	200	3.0	0	0.2	179.2	0.259	−56.3
3	150	1.8	1.2	0.2	173.5	0.280	−38.3
4	150	3.0	0.6	0.1	151.7	0.283	−40.4
5	150	2.4	0	0.3	178.5	0.256	−57.8
6	100	3.0	1.2	0.3	136.5	0.293	−36.8
7	100	1.8	0	0.1	169.9	0.284	−61.5
8	200	1.8	0.6	0.3	173.0	0.266	−42.7
9	100	2.4	0.6	0.2	130.2	0.284	−42.3

*X*	*K*_1_	436.6	516.4	527.6	504.3		
	*K*_2_	503.7	491.4	454.9	482.9		
	*K*_3_	534.6	467.4	492.7	488.0		
	*K*_1_	145.5	172.1	175.8	168.1		
	*K*_2_	167.9	163.8	151.6	161.0		
	*K*_3_	178.3	155.8	164.2	162.7		
	*R*	32.8	16.3	23.7	7.1		

*Y*	*K*_1_	0.861	0.830	0.799	0.829		
	*K*_2_	0.819	0.802	0.833	0.823		
	*K*_3_	0.787	0.835	0.835	0.815		
	*K*_1_	0.287	0.277	0.266	0.276		
	*K*_2_	0.273	0.267	0.278	0.274		
	*K*_2_	136.5	134.9	125.4	136.9		
	*K*_3_	133.8	133.5	109.9	137.3		
	*K*_1_	47.0	47.5	58.5	45.6		
	*K*_2_	45.5	45.0	41.8	45.6		
	*K*_3_	44.6	44.5	36.6	45.8		
	*R*	2.4	3.0	21.9	0.2		

**(a):** 

Ultrasonic intensity (%)	The average diameter (nm)	PDI	Zeta potential (mV)
30	156.5 ± 2.4	0.279 ± 0.011	−39.3 ± 2.8
40	130.7 ± 1.8	0.248 ± 0.055	−39.4 ± 1.6
50	127.0 ± 2.3	0.305 ± 0.006	−41.8 ± 1.3

**(b):** 

Ultrasonic time (min)	The average diameter (nm)	PDI	Zeta potential (mV)
10	131.5 ± 1.7	0.280 ± 0.006	−40.4 ± 2.4
20	105.8 ± 2.8	0.421 ± 0.024	−37.0 ± 1.1
30	110.3 ± 2.5	0.374 ± 0.040	−36.1 ± 2.5

**(c):** 

Ultrasonic cycles (s)	The average diameter (nm)	PDI	Zeta potential (mV)
5-5	139.1 ± 2.4	0.295 ± 0.005	−37.1 ± 1.1
10-10	131.2 ± 0.4	0.281 ± 0.011	−40.1 ± 1.2
15-15	139.5 ± 1.2	0.299 ± 0.006	−36.5 ± 2.2

**(d):** 

Temperature (°C)	The average diameter (nm)	PDI	Zeta potential (mV)
4 ± 4	166.2 ± 4.3	0.270 ± 0.007	−39.4 ± 0.8
25 ± 4	131.0 ± 1.2	0.285 ± 0.006	−40.4 ± 2.0
50 ± 4	118.5 ± 2.0	0.351 ± 0.004	−40.8 ± 2.0

**Table 5:** Results of the stability test of targeted nanoparticle.

Day	The average diameter (nm)	PDI	PH
1	229.4 ± 7.5	0.205 ± 0.014	6.80 ± 0.08
7	216.1 ± 6.4	0.183 ± 0.025	6.42 ± 0.12
15	205.8 ± 4.4	0.184 ± 0.012	6.04 ± 0.04
